# Closer Affiliation Demanded

**Published:** 1897-01

**Authors:** 


### CLOSER AFFILIATION DEMANDED.

Strong in numbers, fully representative of a great sanitary profession; strong in the intellectual character of its members and in full pace with the advance of medical and sanitary science; daily contributing to the progress of the world, the relief of suffering mankind, and the broader domain of the animal world; and equipped to shed light and assistance no longer possible to ignore, we ask at this time, Can any future Medical Congress or Pan-American Convention be complete without a recognized delegation of veterinarians or practitioners in comparative science? The whole domain of our animal friends rises daily in importance as each short cycle of time brings forth the closer relationship of so many forms of disease in man and those of the lower animals. The animal food supply, the chief reliance of man, finds in the veterinarian knowledge and closer observation of the diseases of relative import, not to speak of his special knowledge and training as to their peculiarities, their methods of propagation, the practical methods of control that may be safeguarded through his work and direction. Might we suggest to the newly elected President, Dr. Osgood, that the time is opportune and the need great for a closer affiliation of the students of human and veterinary medical science, and may this not be best accomplished by affiliation in these congresses, where the broad aspects of these important questions are delegated to representative men, many of whom might be veterinarians?

